# Evaluation of duloxetine and innovative pelvic floor muscle training in women with stress urinary incontinence (DULOXING): Study protocol clinical trial (SPIRIT Compliant)

**DOI:** 10.1097/MD.0000000000018834

**Published:** 2020-02-07

**Authors:** Magdalena Hagovska, Jan Svihra

**Affiliations:** a Department of Physiatry, Balneology, and Medical Rehabilitation, Institution - Faculty of Medicine, PJ Safarik University, Kosice; b Department of Urology, Institution - Jessenius Faculty of Medicine, Martin, Comenius University Bratislava, Slovak Republic.

**Keywords:** duloxetine, pelvic floor exercise, stress urinary incontinence

## Abstract

**Introduction:**

There is a lack of published studies about the combination of duloxetine and pelvic floor muscle training (PFMT) in women with stress urinary incontinence (SUI). The aim of our work will be to evaluate the effect of this intervention by assessing whether there is a change in the incontinence episode frequency (IEF), Incontinence Quality of Life (I-QoL), Patient Global Impression of Improvement score (PGI-I) and mean time between voids (MTBV). Combined therapy with duloxetine and PFMT will be compared to duloxetine treatment alone with respect to its efficacy and side effects.

**Methods:**

This study will be a randomized intervention, parallel, multicenter study in collaboration with 45 urological outpatient clinics at the national level. Patients will be assigned in a 1:1 ratio to the experimental and control groups using simple randomization according to odd and even numbers assigned sequentially to the patients at each clinic. The experimental intervention will be 12 weeks. The experimental group will receive oral treatment with duloxetine at a daily dose of 2 × 40 mg and will be required to perform innovative PFMT. The control group will receive the same oral duloxetine treatment (2 × 40 mg a day) but will not perform PMFT. Data will be collected from both groups before intervention and after the 12-week intervention is completed.

**Discussion:**

The study protocol presents the starting points, design and randomization of an interventional multicenter study to monitor the effect of the combination of duloxetine with innovative PFMT compared to duloxetine treatment alone in women with SUI. This study may provide evidence of the efficacy of this combined treatment for SUI and highlight benefits associated with active approaches to treatment through exercise.

**Registration:**

This study was retrospectively registered in the ClinicalTrials.go NCT04140253. Protocol version 1.0. date 11.1.2019.

## 1 Introduction

Urinary incontinence is an involuntary leakage of urine; the most common type is stress urinary incontinence (SUI). SUI is defined as “the complaint of involuntary loss of urine on effort or physical exertion (eg, sporting activities), or on sneezing or coughing”.^[1]^ According to an international consensus, the average prevalence of urinary incontinence is approximately 20% to 30%, and in half of these cases, it is classified as SUI. The estimated prevalence of urinary incontinence in 15-year-old women is 5%. It increases with age to 72% in 60-year-old women.^[2]^ The first-choice treatment to improve SUI is pelvic floor muscle training (PFMT). It is defined as repeated, selective-will contraction and relaxation of specific pelvic floor muscles. It is important to exercise the strength, endurance and relaxation of pelvic floor muscles.^[2,3]^ The PFMT mechanism of action is pelvic floor muscle hypertrophy, which decreases urethral hypermobility and thus reduces urinary incontinence.^[4,5]^

In the literature, there is a lack of published studies that examine a combination of duloxetine and PFMT in women with SUI. In order to ensure the maximum effect and safety of these interventions, it is necessary to continually innovate exercise programs and to update knowledge with relevant guidelines. It is crucial that PFMT is progressive, that is, displays a gradual increase in difficulty. It is also important to ensure that the training program is interesting to keep patients motivated to complete it. In recent years, studies described PFMT using stabilization, so we decided to verify the effect of progressive PFMT with stabilizing exercises in combination with duloxetine.^[6]^

SUI treatment involves increasing the urethral closure pressure by correcting hypermobility, lengthening and strengthening urethral support or enhancing the intrinsic urethral closure mechanism. The hypothesis that elevated serotonin and noradrenaline levels in Onuf's nucleus stimulate the urethral sphincter has been confirmed experimentally.^[7,8]^ Duloxetine is a noradrenaline and serotonin reuptake inhibitor; it increases the synaptic concentrations of both mediators at Onuf's nucleus. Conversely, it minimally affects postsynaptic dopaminergic receptors.^[7]^ Duloxetine causes pudendal nerve stimulation and improves the tone of the sphincter urethra, effects that significantly increases urethral pressure in women with SUI.^[9]^

An initial phase II clinical trial evaluated the efficacy of duloxetine in 48 US centers. Patients aged 18 to 65 years with SUI were included in the trial. Treatment lasted 12 weeks after the screening and dose escalation phases. The efficacy of treatment was assessed by examining the incontinence episode frequency (IEF) each week, the Incontinence Quality of Life questionnaire (I-QoL), the Patient Global Impression of Improvement (PGI-I) and the mean time between voids (MTBV).^[10]^

Duloxetine significantly reduced the number of IEFs per week while improving quality of life according to I-QOL. It also increased the number of patients with positive PGI-I and increased MTBV. The changes were positively dependent on the duloxetine dose. Treatment discontinuation occurred in 5% of patients in the placebo group, 9% of patients taking 20 mg/day duloxetine, 12% of patients taking 40 mg/day duloxetine and 15% of patients taking 80 mg/day duloxetine. The most frequent adverse event was nausea, which occurred in 13% of the patients taking 80 mg/day duloxetine and was the reason for treatment discontinuation in 4.3% of cases.^[10]^

The majority of patients who received 80 mg/day duloxetine who reported nausea continued their treatment. Other undesirable effects included headache, dizziness, dry mouth, insomnia, constipation and nasopharyngitis. These side effects are rare and do not usually cause discontinuation of treatment. Because the 80 mg/day duloxetine dose most efficaciously improved urine leakage while not increasing the number of adverse events, it was recommended for use in a phase III clinical trial.^[10]^

The phase III clinical trial evaluated the clinical efficacy and safety of duloxetine at a daily dose of 80 mg (40 mg twice daily). The clinical trial was double-blinded and placebo-controlled. The clinical file, utilized methods and statistical analysis was similar to those used the phase II clinical trial. The mean percentage change in IEF and mean change in total I-QOL were similarly evaluated. Complete dryness was achieved in 10.5% of duloxetine-treated patients, while only 5.9% of participants in the placebo group achieved dryness. The number of patients who experienced decreased weekly urinary leakage rates (more than half) was significantly higher in the duloxetine group (51%) compared to the placebo group (34%). Nausea was the most common adverse side effect; it occurred in 23% to 28% of the patients taking duloxetine compared to 2% to 7% of participants in placebo group. Although this adverse event occurred in a quarter of all women taking duloxetine, it only caused premature treatment discontinuation in 3% to 6% of cases (it caused premature discontinuation of treatment in 1% of the placebo group). Most women reported nausea when treatment started and throughout the treatment period. Effects only reached a mild or moderate degree (75%–87% of all women with nausea). The nausea was transient in nature and subsided in 40% to 60% of cases after one week and 74% to 86% after 1 month of treatment passed. Thus, after a month of treatment with duloxetine, nausea was only reported in 3% to 7% of all treated women.^[8,11,12]^

Ghoniem et al demonstrated the effect of a 12-week combination of PMFT and duloxetine treatment on women with SUI. The number of IEFs per week, number of pads used, quality of life and subjective evaluation through the PGI-I scale were evaluated. The IEF responder rate in duloxetine vs PFMT group was 56.5% vs 26.1%, median decrease in pads used was 35.3% vs 24.8% and mean increase in I-QoL score was 8.3% vs 7.8%.^[13]^

Maund et al monitored both the benefits and adverse effects associated with duloxetine use for SUI treatment using a meta-analysis. Improvements were reported for reducing SUI symptoms and improving quality of life.^[14]^

### 1.1 Primary goal

The primary goal of this trial is to assess the efficacy of combined duloxetine as comparator and innovative PFMT compared to duloxetine treatment alone in treatment of women with SUI. The primary goal will be monitored based on change in IEFs, according to the International Consultation on Incontinence Questionnaire Short Form (ICIQ-IU SF), over 12 weeks of treatment.

### 1.2 Secondary goals

The secondary goals are to assess the efficacy of the combined duloxetine as comparator and PFMT compared to duloxetine treatment alone. Analysis will include changes in I-QoL over 12 weeks of treatment. Additionally, changes in PGI will be monitored, and any adverse events over 12 weeks of treatment will be recorded.

## 2 Methods

### 2.1 Study design

The Duloxing trial is designed as a randomized controlled intervention and parallel study. The Duloxing study will compare the effect of duloxetine as comparative registered treatment and duloxetine as comparative registered treatment plus innovative PFMT combination therapy within a female population with SUI. Study will examine the effect of authorized registered Duloxetine treatment in the population. The hypothesis states that after 12 weeks of combined treatment, the duloxetine plus PFMT group will have significantly fewer episodes of urine leakage, fewer collection aids and improvements reflected in quality of life assessments compared to the duloxetine alone group. This protocol was approved by the ethics committee of the University Hospital in Martin, Slovakia (number 24012019). All important protocol modifications will be approved by the same ethics committee. Informed consent will be obtain from trial participants by the local clinic of urology. The personal information about potential and enrolled participants will be collected, shared, and protected in concordance with Slovakian low.

### 2.2 Recruitment and consent

The Duloxing study will be performed in 45 community clinics of urology in the Slovakia. The study will use 2 groups in parallel: the experimental group (E) and control group (C). The study will be randomized and controlled in a 1:1 allocation ratio. For the allocation process, sequences will be generated by a computer and designated by a researcher who will not participate further in the study. In this system, the computer generates even and odd patient numbers. Patients with odd numbers will receive duloxetine monotherapy at a daily dose of 2 × 40 mg per tablet for 12 weeks. Patients with even numbers will receive a combined treatment of duloxetine at a daily dose of 2 × 40 mg per tablet for 12 weeks combined with PFMT for the same time period. The generated numbers will be placed in sealed envelopes. Each envelope will contain the group code (E or C). Blinding will prevent intentional selection bias (Table 1).

**Table 1:**
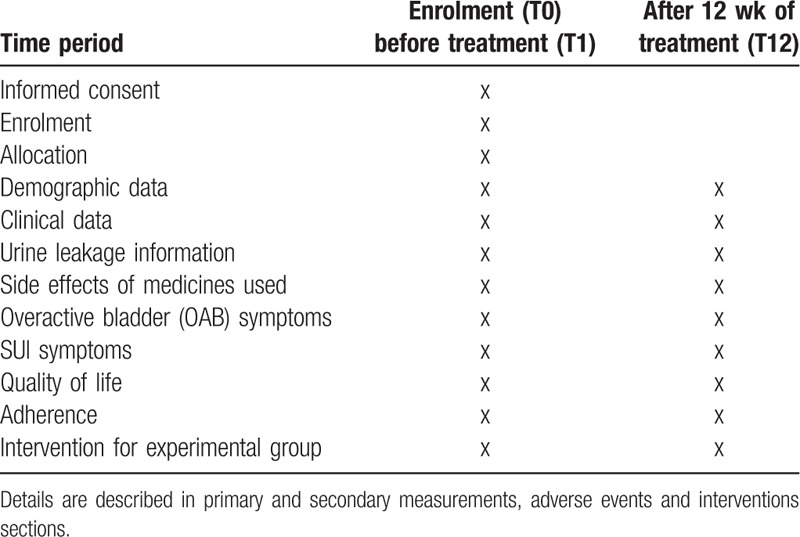
Recruitment, intervention and evaluation plan for experimental and control groups in the Duloxing study.

### 2.3 Sample size

We used power analysis to determine an appropriate sample size; the test strength was set at 0.80 and alpha at 0.05 (type I error). An estimated 63 probands are required for each group (experimental and control). We anticipate a 20% loss in participation and will therefore include a total of at least 158 probands. Based on sample selection, we expect a decrease in incidence after intervention from 10% to 35%. A decrease of more than 50% will be considered a success.

Listed below are the inclusion and exclusion criteria for the DULOXING trial.

### 2.4 Inclusion criteria

1. Woman's willing to provide written informed consent;
2. Women over 18 years old who experience uncomplicated SUI;
3. Score on the International Consultation on Urinary Incontinence Questionnaire ≥14 points;
4. Symptoms of urinary incontinence for at least 3 consecutive months;
5. Have at least seven urinary incontinence episodes per week;
6. Degree of pelvic organ prolapse ≤ stage 2;
7. Willingness to accept the randomization process and fully participate in tests.

### 2.5 Exclusion criteria

1. Recent use of any pharmacologic agent to treat symptoms of urinary incontinence in the past 6 months;
2. History of anti-incontinence surgery in the past 12 months;
3. Use of onabotulinum toxin A for the treatment of urinary incontinence in the past 12 months;
4. History of pelvic prolapse repair or urethral surgery in the past 12 months;
5. History of PFMT in the past 12 months;
6. History of interstitial cystitis or bladder-related pain;
7. Chronic severe constipation;
8. Clinically significant renal or hepatic impairment;
9. Clinically significant heart impairment;
10. Women who are pregnant, lactating or actively trying to become pregnant;
11. Non-compliance with limitation of duloxetine treatment for mixed urinary incontinence;
12. Positive urinary tract infection;
13. Use of rehabilitation aids (pessary, urethral plugs, vaginal beads, et cetera);
14. Use of antidepressant(s);
15. Insufficient understanding of pelvic floor exercises and/or omitting exercises;
16. Participation in any clinical study in the past 6 months.

### 2.6 Interventions

#### 2.6.1 Experimental and control groups

1. Oral administration of daily duloxetine (2 × 40 mg) for 12 weeks.
2. During the screening period, the usual procedure for the indication of duloxetine treatment will be used.
3. Questionnaires will be completed before initiation of duloxetine titration treatment.
4. After a 2-week titration, full treatment in both arms of the study will commence.
5. After 2 weeks of titration treatment, the full dose will be administered. This dose administration will continue until the end of the 12-week treatment for both control and experimental groups.
6. After 12 weeks of full treatment, patients will be required to visit researchers, and they will complete a questionnaire and report adverse effects.
7. At study completion, patients may continue treatment at the discretion of the treating physician.

#### 2.6.2 Experimental group

PFMT with stabilizing exercises (Table 2)

1. Oral administration of treatment with duloxetine at a daily dose of 2 × 40 mg for 12 weeks.
2. PFMT with lumbopelvic stabilization will be performed.
3. PFMT exercises will be performed 5 times a week for 20 to 30 minutes a day, after initial training with a physiotherapist.
  a. Educating probands about anatomy, physiology and pelvic floor muscle functions;
  b. Training pelvic floor muscles in different positions;
  c. Training pelvic floor muscles with lumbopelvic stabilization.

**Table 2:**
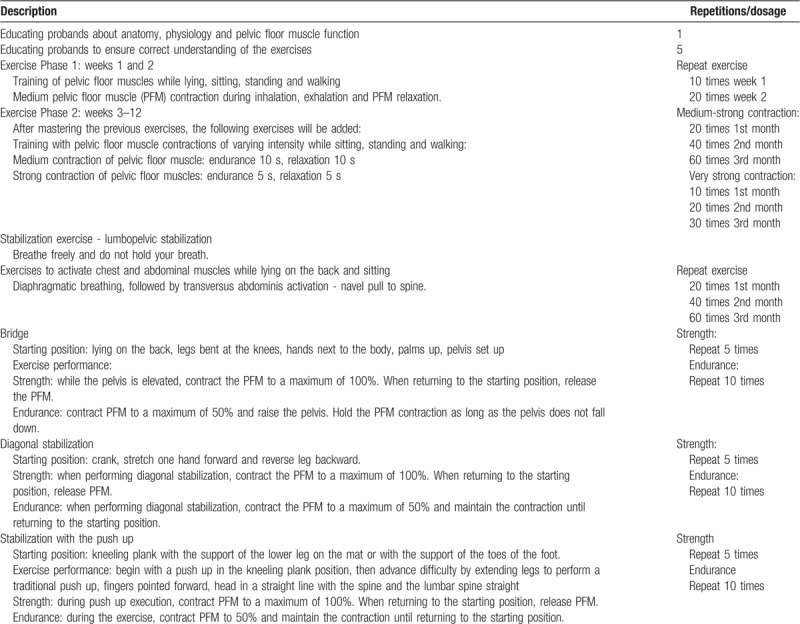
Pelvic floor muscle training exercise protocol.

### 2.7 Outcome measures

#### 2.7.1 Primary measurements

Urine leakage symptoms will be reported according to an assessment from the International Consultation on Incontinence Questionnaire Urinary Incontinence Short Form (ICIQ-UI SF), which was developed by the International Continence Society (ICS). It monitors the frequency and amount of escaped urine in the first 2 questions. The third question assesses how much the urine leakage interferes with the patient's daily life. The ICIQ-UI SF score is the sum of questions: 0 = no leakage, 21 = very severe urine leakage).^[15]^ Cronbach's alpha for the ICIQ-UI SF is 0.95.^[16]^

Symptoms will also be reported using the Overactive Bladder Questionnaire (OAB-q). This questionnaire about urge urinary incontinence symptoms focusses on the symptoms of urge incontinence that occur in the 4 weeks before evaluation. It contains 6 questions with symptom scores that range from 0 (none) 100 (maximum) symptoms and 13 questions that assess quality of life. A score of 100 corresponds to the best quality of life and 0 the worst.^[17,18]^ Cronbach's alpha for the OAB-q is 0.90.^[19]^

#### 2.7.2 Secondary measurements

##### 2.7.2.1 Change in quality of life according to I-QoL

I-QoL is a self-assessment scale for evaluating the quality of life of urinary incontinence patients. It is divided into three subscales: avoidance and limiting behavior, psychosocial impact and social embarrassment. It contains 22 questions (0 = worst quality of life, 100 = best quality of life). Cronbach's alpha for the I-QoL is 0.91 to 0.96.^[20–22]^

##### 2.7.2.2 PGI-I score

The PGI evaluates the status of urination problems compared to the condition before a patient started treatment in the study. Patient impressions are evaluated according to the following scores: 1 = much better; 2 = quite better; 3 = a little better; 4 = no change; 5 = a little worse; 6 = a lot worse; 7 = definitely worse.^[23]^

Comparison of the combined duloxetine and PFMT therapy to duloxetine treatment alone will be evaluated according to efficacy and side effects.

#### 2.7.3 Adverse events - harms

The adverse events in connection with duloxetine use will be investigated. These effects include nausea, fatigue, insomnia, dry mouth, constipation, impaired consciousness, dizziness, headache, diarrhea, double vision depression, and abdominal pain.

### 2.8 Adherence

Eligibility, cooperation and security will be recorded during recruitment and study implementation. The lead researcher, in collaboration with urologists, will register appropriate patients from all databases in outpatient registries. The number of suitable patients from the total patients considered will be recorded. During the intervention period, collaboration and adherence as well as treatment discontinuation will be recorded. Adherence to medication dosage and exercise treatment specifications will be required.

### 2.9 Data analysis

Descriptive and inference statistics will be used to analyze the data. Unpaired *t* tests will be used to compare the experimental and control groups before training. We expect that our data will be normally distributed. Differences between the control and experimental groups before and after the intervention will be evaluated with a general linear model (GLM) and mixed design analysis of variance (ANOVA) using repeated measurements with the Greenhouse-Geisser correction. The significance level will be set at 95%, and *P* < .05 will be considered significant. Effect size (ES) will be calculated based on partial eta squared (η^2^). According to Cohen,^[24]^ the small, medium and large ANOVA effect sizes (η^2^) will be classified as: 0.00 to 0.003, no effect; 0.010 to 0.039, small; 0.060 to 0.110, medium; 0.140 to 0.200, big. Calculations will be made in IBM SPSS 22 Windows (IBM, Chicago, IL).

### 2.10 Monitoring

The study will be controlled by an independent individual. The Commission will determine the authority of the study. JS's Principal Investigator will be responsible for organizing research activities and communicating with patients, associates and partners. The Co-Investigator will manage central randomization, project and ethical standards and data collection, protection, entry, storage, and processing. Explanation of the examination and exercise in the study will be performed by 45 members of the research team. During study interim analyses will be done by the data management team.

## 3 Discussion

The study protocol presents the starting points and design of a randomized-intervention, multicenter study with the aim of evaluating the effect of the combination duloxetine and PFMT therapy versus duloxetine treatment alone in women with SUI. The study may provide evidence of the efficacy of combined treatment for SUI and indicate an active approach for the treatment of SUI through exercise.

The strength of the study is it promotes an innovative, non-invasive and conservative treatment of strengthening pelvic floor muscles with stabilization exercises in addition to duloxetine treatment. We will use 3 standardized measuring tools: ICIQ-UI SF, OAB-q-short version and I-QoL. Both groups will receive daily oral 80 mg duloxetine (2 × 40 mg), and an innovative PFMT will be added in the experimental group. Interventions will last for 12 weeks, and will consist of exercise 5 times a week for 30 minutes. Education of probands regarding anatomy, physiology, and pelvic floor muscles—so that they properly understand the impact of exercises—will be done by a physiotherapist in collaboration with a nurse 5 times. Subsequently, exercises will be performed at home, and a diary control will be used to ensure compliance.

Based on similar studies, we expect good patient adherence, a low level of patient dropout and agreement with the study protocol. Patients who will be assigned to a duloxetine-only group will be offered the opportunity to exercise after of the 12-week treatment. We expect a 20% subject dropout. The success of our treatment will be a decline of more than 50% in the difficulties associated with SUI symptoms.

## Acknowledgments

We thank the doctors from urological clinics for their cooperation in the study.

## Author contributions

JS is responsible for study design and methodology. MH is responsible for writing articles and preparing educational materials for exercise. JS and MH are responsible for coordinating center, steering committee, endpoint adjudication committee and data management. JS and MH will have access to the final trial dataset. Both authors MH, JS have read and approved the final manuscript.

Jan Svihra orcid: 0000-0003-0164-6359.
